# Supplementary material for: Time‑resolved multi-omic analysis of paclitaxel exposure in human iPSC‑derived sensory neurons unveils mechanisms of chemotherapy‑induced peripheral neuropathy
Source: Cell Death Dis. 2026 Feb 10;17(1):211. doi: 10.1038/s41419-026-08445-2 (PMC12921266; doi:10.1038/s41419-026-08445-2)
Supplement: Supplementary file 15 — Dataset 1. [file 41419_2026_8445_MOESM15_ESM.zip › CDD_Open_Data/04_RNA_Seq/Timeline_panel_plots_etc_2023-01-23_1609.zip!/Panel_Plots_etc/Evidence_Plots/24h/evidence_plot_standard contrasts_msigdb_hallmark_24h_PTX_vs_DMSO_ID4_M5953_HALLMARK_KRAS_SIGNALING_UP.pdf]

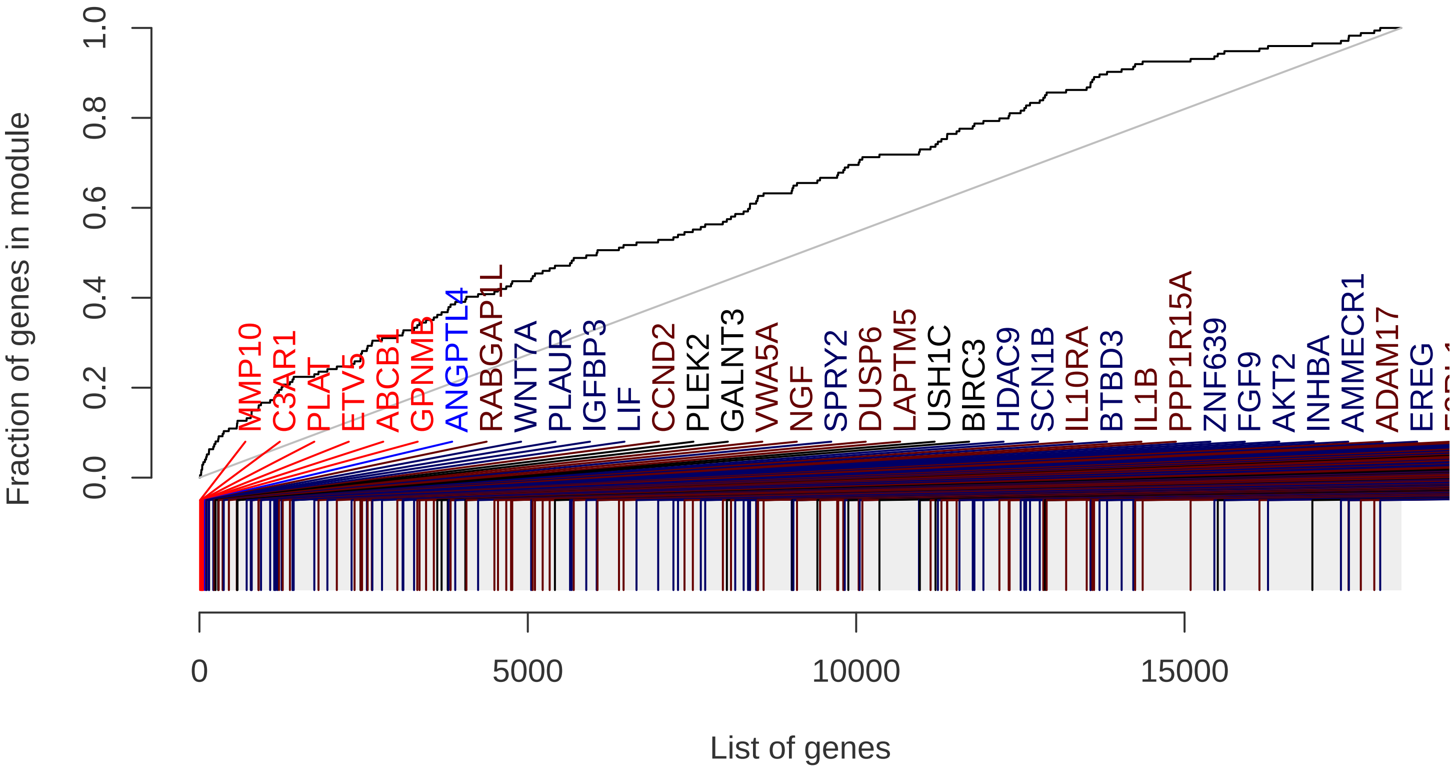
Fraction of genes in module
0.0
0.2
0.4
0.6
0.8
1.0
MMP10
C3AR1
PLAT
ETV5
ABCB1
GPNMB
ANGPTL4
RABGAP1L
WNT7A
PLAUR
IGFBP3
LIF
CCND2
PLEK2
GALNT3
VWA5A
NGF
SPRY2
DUSP6
LAPTM5
USH1C
BIRC3
HDAC9
SCN1B
IL10RA
BTBD3
IL1B
PPP1R15A
ZNF639
FGF9
AKT2
INHBA
AMMECR1
ADAM17
EREG
0
5000
10000
15000
List of genes
